# Ovulation Statuses of Surrogate Gilts Are Associated with the Efficiency of Excellent Pig Cloning

**DOI:** 10.1371/journal.pone.0142549

**Published:** 2015-11-13

**Authors:** Yanjun Huan, Kui Hu, Bingteng Xie, Yongqian Shi, Feng Wang, Yang Zhou, Shichao Liu, Bo Huang, Jiang Zhu, Zhongfeng Liu, Yilong He, Jingyu Li, Qingran Kong, Zhonghua Liu

**Affiliations:** College of Life Science, Northeast Agricultural University, Harbin, Heilongjiang Province, China; Guangzhou Institute of Biomedicine and Health, CHINA

## Abstract

Somatic cell nuclear transfer (SCNT) is an assisted reproductive technique that can produce multiple copies of excellent livestock. However, low cloning efficiency limits the application of SCNT. In this study, we systematically investigated the major influencing factors related to the overall cloning efficiency in pigs. Here, 13620 cloned embryos derived from excellent pigs were transferred into 79 surrogate gilts, and 119 live cloned piglets were eventually generated. During cloning, group of cloned embryos derived from excellent Landrace or Large white pigs presented no significant differences of cleavage and blastocyst rates, blastocyst cell numbers, surrogate pregnancy and delivery rates, average numbers of piglets born and alive and cloning efficiencies, and group of 101–150, 151–200 or 201–250 cloned embryos transferred per surrogate also displayed a similar developmental efficiency. When estrus stage of surrogate gilts was compared, group of embryo transfer on Day 2 of estrus showed significantly higher pregnancy rate, delivery rate, average number of piglets born, average alive piglet number or cloning efficiency than group on Day 1, Day 3, Day 4 or Day 5, respectively (P<0.05). And, in comparison with the preovulation and postovulation groups, group of surrogate gilts during periovulation displayed a significantly higher overall cloning efficiency (P<0.05). Further investigation of surrogate estrus stage and ovulation status displayed that ovulation status was the real factor underlying estrus stage to determine the overall cloning efficiency. And more, follicle puncture for preovulation, not transfer position shallowed for preovulation or deepened for postovulation, significantly improved the average number of piglets alive and cloning efficiency (P<0.05). In conclusion, our results demonstrated that ovulation status of surrogate gilts was the fundamental factor determining the overall cloning efficiency of excellent pigs, and follicle puncture, not transfer position change, improved cloning efficiency. This work would have important implications in preserving and breeding excellent livestock and improving the overall cloning efficiency.

## Introduction

Somatic cell nuclear transfer (SCNT) is an assisted reproductive technique in which a somatic cell is transferred into an enucleated oocyte to generate a new individual, genetically identical to donor cell, and cloned animals can share the characteristics of the donor breed [1–3]. Thus, SCNT can produce multiple copies of excellent livestock and has an attractive prospect to accelerate the large-scale multiplication of superior genotypes [4].

Since the first cloned pigs were successfully generated [5–7], SCNT has been used to amplify genetically superior pigs, and some cloned pigs with superior genotypes have been produced [3]. However, to date, the overall cloning efficiency is still low, and this limits the extensive application of excellent pigs [8].

It is known that cloning is a complex multistep procedure, and many factors, such as donor cell type, number of cloned embryos transferred per surrogate, embryo transfer method and ovulation status of surrogates, etc, have been shown to influence the overall cloning efficiency [8–10], thus, to investigate and optimize these factors may improve the overall cloning efficiency and facilitate the wide application of excellent pigs.

Previous studies have applied different approaches to improve pig cloning efficiency and suggested that selecting a suitable donor cell type, transferring an appropriate number of cloned embryos per surrogate or coordinating the synchronization of cloned embryos and surrogates, etc, could increase the success rate of cloned piglets [11–15]. However, these studies are usually done unsystematically, and reports on the systematical investigation into the factors influencing the overall cloning efficiency are rare. Also, studies on excellent pig cloning and analysis of the related influencing factors are very few. Here, in view of the vital role of excellent pigs in swine industry and to accelerate the large-scale multiplication of of superior genotypes, we systematically investigated the effects of donor cell type, number of cloned embryos transferred per surrogate, estrus stage of surrogate gilts, surrogate ovulation status and embryo transfer manners on excellent pig cloning. In this study, we successfully generated 119 alive cloned piglets with superior genotypes, and found that ovulation status of surrogate gilts significantly influenced the overall cloning efficiency of excellent pigs, and follicle puncture, not transfer position change, improved cloning efficiency. This work would have important implications in preserving and breeding excellent livestock and improving the overall cloning efficiency.

## Materials and Methods

Chemicals were purchased from Sigma Aldrich Corporation (St. Louis, MO, USA), and disposable and sterile plasticware was obtained from Nunclon (Roskilde, Denmark), unless otherwise stated.

All the treatments of pigs were approved by Animal Care and Use Commission of Northeast Agricultural University, according to animal welfare laws, guidelines and policies. All surgery was performed under sodium pentobarbital anaesthesia, and all efforts were made to minimize suffering.

### Porcine adult fibroblast (PAFs) culture

PAFs were isolated from the ears of Landrace (L-PAFs) or Large white (LW-PAFs) breeding boars under sodium pentobarbital anaesthesia with the good reproductive performance in Harbin Sanyuan Livestock Industry Co., Ltd., located in Harbin city, Heilongjiang province [16]. After removal of skin tissues and gristle, the remaining tissues were finely minced into pieces, digested with 0.25% trypsin-0.04% ethylenediaminetetraacetic acid solution (GIBCO), and dispersed in high glucose enriched Dulbecco’s modified Eagle’s medium (DMEM, GIBCO) containing 10% fetal bovine serum (FBS, GIBCO) and 1% penicillin-streptomycin (GIBCO). Then, the dispersed cells were centrifuged, resuspended and cultured in DMEM. Until confluence, PAFs were digested, centrifuged, resuspended in FBS containing 10% dimethyl sulfoxide and stored in liquid nitrogen until use. Prior to SCNT, PAFs were thawed, cultured and subsequently used in 3–5 passages.

### Oocyte collection and in vitro maturation

Oocyte maturation has been described previously [17]. Briefly, porcine ovaries were collected from a slaughterhouse of Harbin Dazhong Roulian Food Co., Ltd., located in Harbin city, Heilongjiang province. Just after ovary exposure, they were placed into physiological saline with antibiotics at 37°C and transported to the laboratory. Follicles were aspirated, and follicular contents were washed with HEPES buffered Tyrode's lactate. Cumulus-oocyte complexes (COCs) were recovered, washed and cultured in maturation medium. After 42 h, COCs were vortexed in hyaluronidase to remove cumulus cells. Only oocytes with a visible polar body, regular morphology and homogenous cytoplasm were used.

### SCNT and embryo culture

The procedure for SCNT has been described in our previous reports [18–20]. Briefly, matured oocytes and PAFs were placed into manipulation medium. After enucleation, donor cells were placed into the perivitelline space. Fusion and activation of the cell-cytoplast complexes were induced by electroporation, and the fusion rate was confirmed by microscopic examination. Reconstructed embryos were cultured in porcine zygotic medium 3 for subsequent development. The cleavage and blastocyst rates were evaluated at 48 h and 156 h, respectively.

### Blastocyst cell number

Cloned blastocysts at 156 h postactivation were treated with Tyrode's acidic solution to remove zona pellucida, fixed in 4% paraformaldehyde for 30 min, and stained in Dulbecco's phosphate buffered saline containing 10 μg/ml Hoechst 33342 for 5 min in dark. After staining, cloned embryos were washed, transferred into antifade mounting medium (Beyotime) on the slides and covered with cover glass, and the edges were sealed with nail polish. Then, blastocyst cell number was examined under ultraviolet light of fluorescence microscope.

### Embryo transfer, pregnancy diagnosis and birth

Embryo transfer has been described [16]. Cloned embryos were cultured at about 0:00 am on the day of embryo transfer. After 12 h culture, cloned embryos with good morphology were selected, kept in manipulation medium and transported to the experiment farm in a portable incubator at 39°C in 1 h. During laparotomy, cloned embryos were loaded into a sterilized straw to be transferred into a surrogate. As the surrogate, a gilt with natural estrus and good health was selected. Usually, surrogate gilts were checked for estrus at 6:00 am and 8:00 pm daily, and only surrogate gilts with a stead standing response at 8:00 pm were chosen, so Day 0 of estrus was 8:00 pm when the surrogate chosen. Day 1 referred to 2:00 pm just after the day of surrogate selection, Day 2 was 24 h after Day 1, and Day 3, Day 4 and Day 5 were successively 24 h later. When embryo transfer, Day 1–5 surrogate gilts were anaesthetized, and the oviduct was exposed by cutting an about 8 cm incision along the abdomen midline between the last two pairs of teats under sodium pentobarbital anaesthesia. After ovulation status examination (preovulation, follicles large developed but not ovulated; periovulation, follicles partly ovulated and postovulation, follicles all ovulated), the embryo-loaded straw was inserted to the oviduct ampulla, which is about 10 cm (approximately 2/3 length of oviduct) from umbrella, and 101–250 cloned embryos were injected. Pregnancy diagnosis was performed by ultrasonography on the 30th day after embryo transfer. When birth, the surrogates were injected with prostaglandin if spontaneous farrowing did not occur, and after 24 h, the surrogates delivered vaginally under supervision, or caesarean section was conducted to deliver cloned piglets if the surrogates still did not start to farrow. When birth, the number of cloned piglets was examined. After all surgical operation and piglet birth, pigs were monitored every 2 h.

### Statistical analysis

If one surrogate became pregnant or deliveried, the percentage of pregnancy or delivery was 100%, respectively, otherwise, 0%. Differences in data (mean ± SEM) were analyzed with SPSS statistical software. Statistical analysis of all data were analyzed through a general linear model. For all analyses, differences were considered to be statistically significant when P<0.05.

## Results

### General information

During excellent pig cloning, 13620 cloned embryos were transferred into 79 surrogate gilts, 50 surrogates (63.29%) became pregnant, 43 surrogates (54.43%) gave birth to offsprings and 119 of 177 cloned piglets born were alive when birth. And, cloning efficiency (total number of live piglets/total number of transferred embryos) was 0.93%.

**Fig 1:**
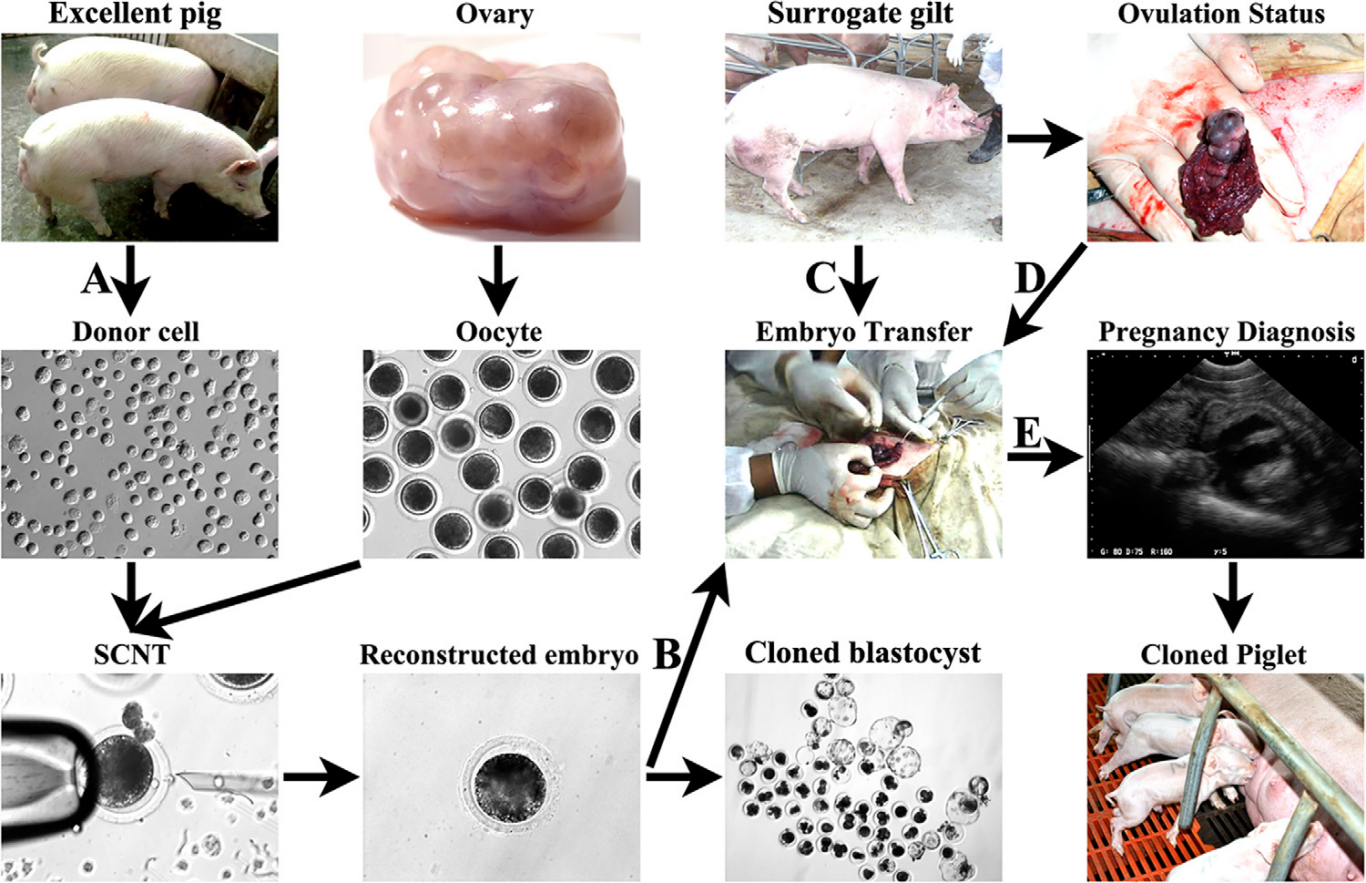
The studied influencing factors related to excellent pig cloning. A, donor cells derived from excellent Landrace or Large white breeding boars, B, 101–150, 151–200 or 201–250 cloned embryos transferred per surrogate, C, Day 1–5 of surrogate estrus, D, ovulation statuses including preovulation, periovulation and postovulation, and E, transfer position change or follicle puncture. Major factors including donor cell type, number of cloned embryos transferred per surrogate, surrogate estrus stage, ovulation status and embryo transfer manners were systematically investigated during excellent pig cloning.

Then, we systematically investigated the effects of donor cell type, number of cloned embryos transferred per surrogate, surrogate estrus stage, ovulation status and embryo transfer manners on pig cloning (Fig 1). Here, we displayed the detail number of surrogate gilts applied in the experiment design (S1 Fig). Among 79 surrogate gilts, 57 surrogates were used to assess the effect of normal transfer (no operation to transfer position and follicle) including donor cell type, number of cloned embryos transferred, estrus stage and ovulation status, and 58 surrogates containing 15 preovulation normally transferred, 21 postovulation normally transferred, 13 transfer position changed and 9 follicle punctured were investigated to analyze the effect of transfer manner. Notably, the extra 36 surrogates were the 15 preovulation and 21 postovulation surrogates in the normal transfer group, and also the control in the transfer manner changed group. Then, a total of 79 surrogate gilts were investigated to assess the effect of individual factor on the overall cloning efficiency.

### No significant effect of donor cell type on the overall cloning efficiency

When L-PAFs and LW-PAFs were used as donor cells, no significant differences of rates of fusion, cleavage and blastocyst and blastocyst cell number were observed (Table A in S1 File and S2 Fig). After cloned embryos transferred into the surrogates (Table 1 and S3 Fig), L-PAFs and LW-PAFs groups did not show significant differences of pregnancy and delivery rates, numbers of born and live piglets per surrogate and cloning efficiency. Thus, L-PAFs and LW-PAFs groups exhibited a similar developmental competence.

**Table 1:** Effect of donor cell types on in vivo development of cloned embryos.

Cell types	No. surrogates (embryos transferred)	No. pregnancy (%)	No. delivery (%)	No. piglets (mean ± SEM)	No. piglets alive (mean ± SEM)	Cloning efficiency (%)
**L-PAFs**	11 (1930)	8 (72.73 ± 14.60)	7 (63.64 ± 15.13)	27 (2.46 ± 0.87)	18 (1.64 ± 0.60)	0.99 ± 0.38
**LW-PAFs**	46 (7905)	29 (63.04 ± 7.14)	26 (56.52 ± 7.40)	113 (2.46 ± 0.43)	74 (1.61 ± 0.29)	0.97 ± 0.19

L-PAFs and LW-PAFs groups displayed no significant differences of in vivo development.

The rates of pregnancy and delivery and the average numbers of piglets born and alive were based on the number of surrogates.

Cloning efficiency was calculated by total number of live cloned piglets/total number of transferred cloned embryos.

### Number of cloned embryos transferred per surrogate had no significant effect on the overall cloning efficiency

We divided the number of transferred embryos into three experimental groups: 101–150, 151–200 and 201–250 (Table 2), and found that rates of pregnancy and delivery and numbers of born and live piglets per surrogate in the 101–150 group were lower in comparison with those in the 151–200 or 201–250 group, seeming that the development of cloned embryos could be associated with the number of embryos transferred. However, cloning efficiency of the 101–150 group was higher than those of the 151–200 and 201–250 groups, and rates of pregnancy and delivery, numbers of born and live piglets per surrogate and cloning efficiency were not significantly different among these three groups. These results suggested that, within the examined number range, the number of cloned embryos transferred per surrogate was not a major factor determining the overall cloning efficiency.

**Table 2:** Effect of transferred cloned embryo number per surrogate on in vivo development of cloned embryos.

Number of embryos transferred	No. surrogates (transferred embryos)	No. pregnancy (%)	No. delivery (%)	No. piglets (mean ± SEM)	No. piglets alive (mean ± SEM)	Cloning efficiency (%)
**101–150**	19 (2475)	11 (57.90 ± 11.19)	10 (52.63 ± 11.60)	37 (1.95 ± 0.66)	26 (1.37 ± 0.46)	1.07 ± 0.29
**151–200**	22 (3820)	15 (68.18 ± 10.40)	13 (59.09 ± 10.78)	52 (2.36 ± 0.61)	36 (1.64 ± 0.42)	0.99 ± 0.27
**201–250**	16 (3540)	11 (68.75 ± 12.19)	10 (62.50 ± 12.64)	51 (3.19 ± 0.72)	30 (1.88 ± 0.50)	0.85 ± 0.32

No significant differences of in vivo development were observed among the examined numbers of cloned embryos transferred

The rates of pregnancy and delivery and the average numbers of piglets born and alive were based on the number of surrogates.

Cloning efficiency was calculated by total number of live cloned piglets/total number of transferred cloned embryos.

### Estrus stage of surrogate gilts influenced the overall cloning efficiency

Surrogate status is also considered to be a major factor influencing the successful generation of SCNT pigs. The result of surrogate estrus stage (Table 3) showed that the Day 2 group exhibited a significantly higher pregnancy rate compared with the Day 1, Day 3 and Day 5 groups, a significantly higher delivery rate in comparison with the Day 5 group, a significantly higher number of piglets per surrogate compared with the Day 4 and Day 5 groups and a significantly higher average number of piglets alive and cloning efficiency in comparison with the Day 3, Day 4 and Day 5 groups (P<0.05). And, the Day 1 group also showed higher rates of pregnancy and delivery, numbers of born and live piglets per surrogate and cloning efficiency than those of the Day 3, Day 4 or Day 5 group, respectively. These results revealed that surrogate estrus stage could be a major factor affecting the overall cloning efficiency, and surrogate gilts with estrus on Day 2 were highly suitable as recipients. Actually, Day 2 of estrus has lasted about 42 h when cloned embryos (14 h postactivation) were transferred, meaning that the optimal synchronization was that estrus stage of surrogates was 28 h prior to the activation time of cloned embryos. Thus, appropriate estrus stage of surrogates for a certain stage embryos can be the guarantee for the successful generation of SCNT pigs.

**Table 3:** Effect of surrogate estrus stage on in vivo development of cloned embryos.

Day of estrus	No. surrogates (transferred embryos)	No. pregnancy (%)	No. delivery (%)	No. piglets (mean ± SEM)	No. piglets alive (mean ± SEM)	Cloning efficiency (%)
**Day 1**	11 (1810)	6 (54.55 ± 13.83)^a^	6 (54.55 ± 14.45)^a^ ^b^	26 (2.36 ± 0.82)^a^ ^b^	18 (1.64 ± 0.56)^a^ ^b^	1.09 ± 0.35^a^ ^b^
**Day 2**	20 (3320)	18 (90.00 ± 10.26)^b^	16 (80.00 ± 10.72)^a^	75 (3.75 ± 0.61)^a^	51 (2.55 ± 0.42)^a^	1.58 ± 0.26^a^
**Day 3**	14 (2505)	7 (50.00 ± 12.26)^a^	7 (50.00 ± 12.81)^a^ ^b^	29 (2.07 ± 0.73)^a^ ^b^	17 (1.21 ± 0.50)^b^	0.64 ± 0.31^b^
**Day 4**	7 (1215)	4 (57.14 ± 17.34)^a^ ^b^	3 (42.86 ± 18.11)^a^ ^b^	8 (1.14 ± 1.03)^b^	5 (0.71 ± 0.70)^b^	0.37 ± 0.44^b^
**Day 5**	5 (985)	2 (40.00 ± 20.51)^a^	1 (20.00 ± 21.43)^b^	2 (0.40 ± 1.22)^b^	1 (0.20 ± 0.83)^b^	0.08 ± 0.52^b^

Group of surrogate gilts with estrus on Day 2 displayed significantly higher rates of pregnancy and delivery, average numbers of piglets born and alive, and cloning efficiency.

The rates of pregnancy and delivery and the average numbers of piglets born and alive were based on the number of surrogates.

Cloning efficiency was calculated by total number of live cloned piglets/total number of transferred cloned embryos.

^a,b^Values in the same column with different superscripts differ significantly (P < 0.05).

### Ovulation status of surrogate gilts determined the overall cloning efficiency

**Fig 2:**
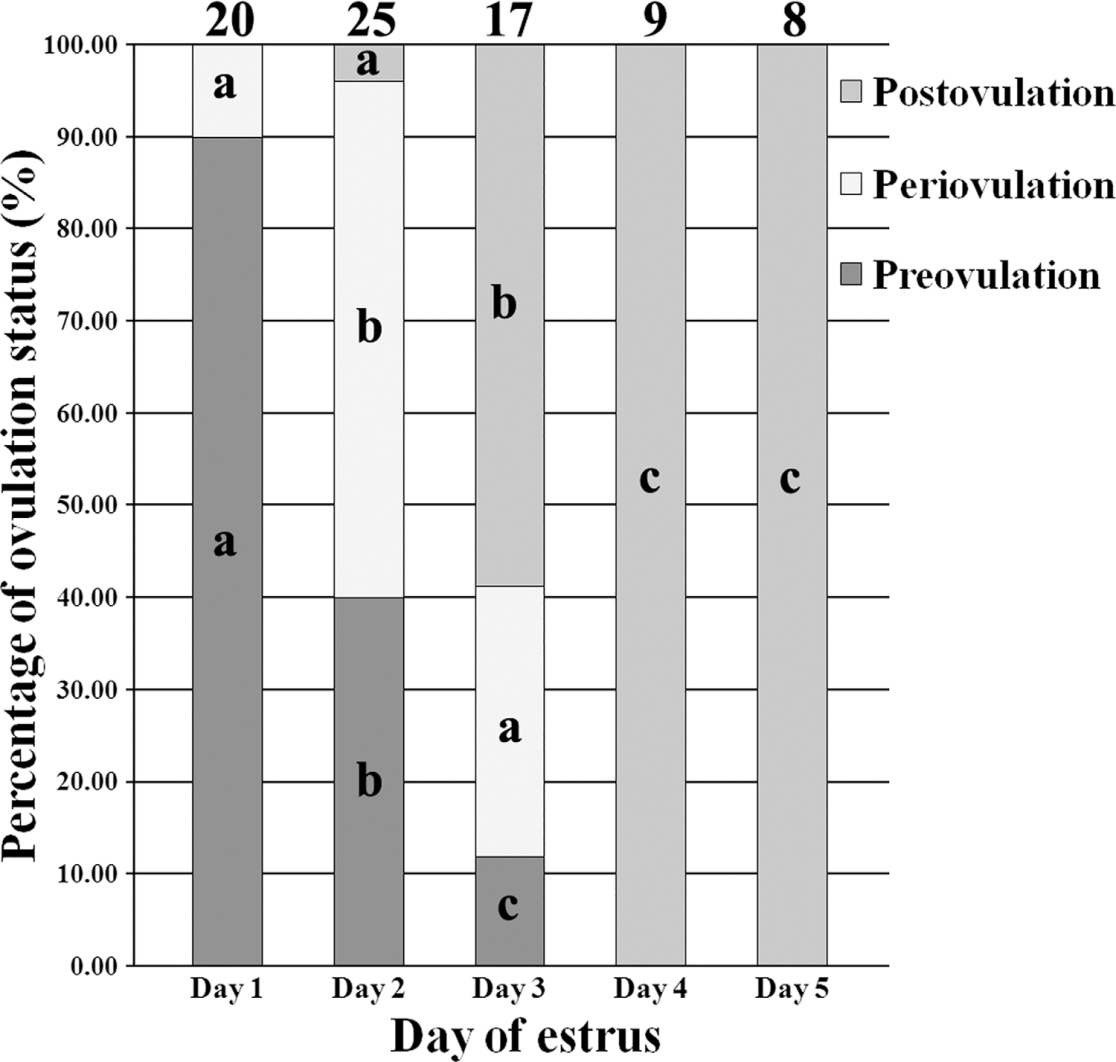
The percentage of surrogate ovulation statuses on Day 1–5 of estrus. The proportions of preovulation, periovulation and postovulation on Day 1–5 of estrus. Preovulation and periovulation mainly occurred on Day 1 and Day 2, respectively, and all the surrogates were under postovulation on Day 4 and Day 5 of estrus. The number of surrogates detected was on the top of column chart. ^a-c^Values for a given status with different superscripts differ significantly (P<0.05).

**Fig 3:**
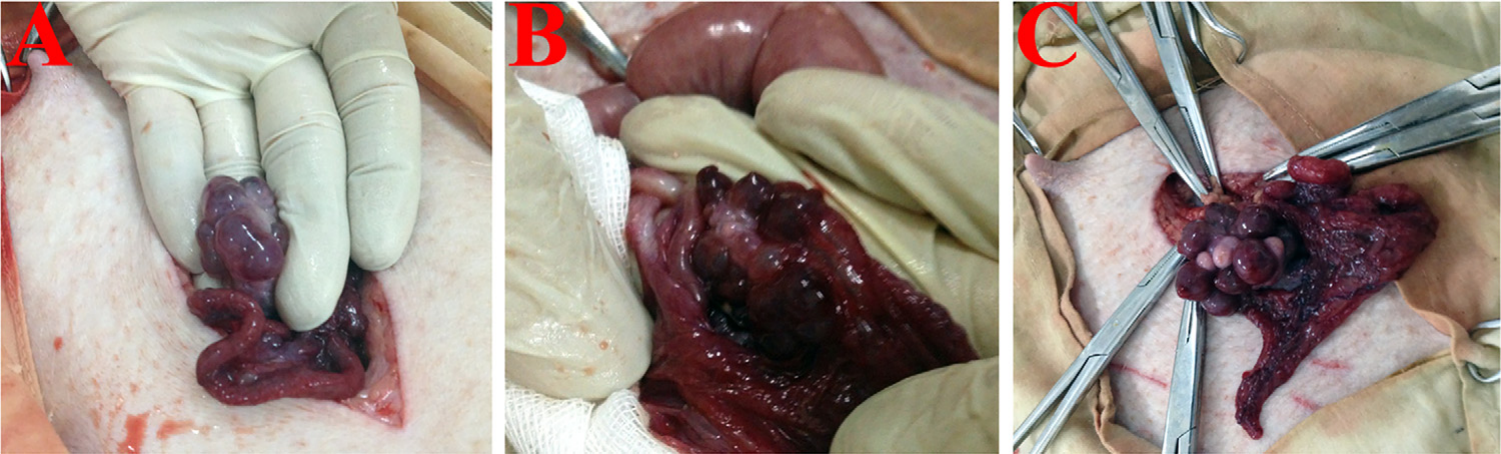
Ovulation statuses of surrogate gilts when cloned embryos transferred. A, preovulation, follicles large developed but not ovulated; B, periovulation, follicles partly ovulated; and C, postovulation, follicles all ovulated. Surrogate gilts during periovulation were suitable for pig cloning.

Generally, ovulation status of surrogates is associated with estrus stage. When cloned embryos were transferred (Fig 2), the Day 1 group, 18 h after estrus, showed 90.00% surrogates under preovulation and 10% periovulation, the Day 2 or Day 3 group (42 h or 66 h after estrus) displayed 40.00% or 11.76% surrogates preovulation, 56.00% or 29.41% periovulation and 4.00% or 58.82% postovulation, respectively, and the Day 4 and Day 5 groups (90 and 114 h after estrus) were all under postovulation, indicating that ovulation status could be a factor related to the overall cloning efficiency. When the effect of ovulation statuses was investigated (Table 4 and Fig 3), significantly higher rates of pregnancy and delivery, numbers of born and live piglets per surrogate and cloning efficiency were observed in the periovulation group compared with the preovulation or postovulation group (P<0.05), and the postovulation group displayed the lowest overall cloning efficiency, suggesting that ovulation status could be also an important factor determining the overall cloning efficiency. Further investigation of the mixed effects of estrus stage and ovulation status displayed that ovulation status was the real factor underlying estrus stage to determine the overall cloning efficiency (Table B in S1 File). And, for surrogates under periovulation in the Day 2 group (Table C in S1 File), both the rates of pregnancy and delivery were 100%, numbers of piglets born and alive per surrogate was 5.00 and 3.50, respectively, and cloning efficiency reached 2.20%. In all, surrogate ovulation status was the fundamentally decisive factor influencing the overall cloning efficiency, and selecting gilts during periovulation as surrogates to transfer cloned embryos can facilitate the synchronization of cloned embryos and surrogates, thereby, resulting in the high overall cloning efficiency.

**Table 4:** Effect of surrogate ovulation status on in vivo development of cloned embryos.

Ovulation status	No. surrogates (transferred embryos)	No. pregnancy (%)	No. delivery (%)	No. piglets (mean ± SEM)	No. piglets alive (mean ± SEM)	Cloning efficiency (%)
**Preovulation**	15 (2250)	9 (60.00 ± 11.41)^a^	8 (53.33 ± 11.02)^a^	25 (1.67 ± 0.60)^a^	15 (1.00 ± 0.39)^a^	0.59 ± 0.25^a^
**Periovulation**	21 (3450)	19 (90.48 ± 9.65)^b^	19 (90.48 ± 9.31)^b^	97 (4.62 ± 0.51)^b^	68 (3.24 ± 0.33)^b^	2.02 ± 0.21^b^
**Postovulation**	21 (3835)	9 (42.86 ± 9.65)^a^	6 (28.57 ± 9.31)^a^	18 (0.86 ± 0.51)^a^	9 (0.43 ± 0.33)^a^	0.21 ± 0.21^a^

The periovulation group showed significantly higher rates of pregnancy and delivery, average numbers of piglets born and alive, and cloning efficiency.

The rates of pregnancy and delivery and the average numbers of piglets born and alive were based on the number of surrogates.

Cloning efficiency was calculated by total number of live cloned piglets/total number of transferred cloned embryos.

^a,b^Values in the same column with different superscripts differ significantly (P < 0.05).

### Follicle puncture, not transfer position change, improved cloning efficiency

It is considered that low cloning efficiency in the preovulation or postovulation group could be probably due to the disrupted synchronization of cloned embryos and surrogates. Here, to coordinate the synchronization, transfer position was shallowed or deepened about 2 cm from the umbrella for the preovulation or postovulation group (Table 5 and S4 Fig), respectively, however, neither shallowing nor deepening transfer position improved the rates of pregnancy and delivery, numbers of born and live piglets per surrogate or cloning efficiency, and deepening transfer position resulted in the even lower overall cloning efficiency, suggesting that regulating the synchronization of cloned embryos and surrogates was not simply to change transfer position. When follicles were punctured for the preovulation group (Table 5 and S4 Fig), a significantly higher average number of cloned piglets alive per surrogate and cloning efficiency were observed in comparison with the normal preovulation group (P<0.05), indicating that, to some extent, follicle puncture could rescue the physiological defect in the normal preovulation group and adjust cloned embryos to adapt the oviduct environment. Thus, follicle puncture, not transfer position change, improved the successful generation of cloned piglets.

**Table 5:** Effect of transfer manners on in vivo development of cloned embryos.

Transfer manners	No. surrogates (transferred embryos)	No. pregnancy (%)	No. delivery (%)	No. piglets (mean ± SEM)	No. piglets alive (mean ± SEM)	Cloning efficiency (%)
**Normal transfer for preovulation**	15 (2550)	9 (60.00 ± 12.66)	8 (53.33 ± 13.36)	25 (1.67 ± 0.55)	15 (1.00 ± 0.36)^a^	0.60 ± 0.23^a^
**Follicle puncture for preovulation**	9 (1545)	7 (77.78 ± 16.34)	6 (66.67 ± 17.25)	25 (2.78 ± 0.70)	20 (2.22 ± 0.46)^b^	1.44 ± 0.30^b^
**Transfer position shallowed for preovulation**	6 (1010)	4 (66.67 ± 20.01)	3 (50.00 ± 21.13)	9 (1.50 ± 0.86)	6 (1.00 ± 0.56)^a^ ^b^	0.69 ± 0.37^a^ ^b^
**Normal transfer for postovulation**	21 (3835)	9 (42.86 ± 10.97)	6 (28.57 ± 9.71)	18 (0.86 ± 0.36)	9 (0.43 ± 0.19)	0.21 ± 0.10
**Transfer position deepened for postovulation**	7 (1230)	2 (28.57 ± 19.00)	1 (14.29 ± 16.81)	3 (0.43 ± 0.62)	1 (0.14 ± 0.33)	0.08 ± 0.17

Follicle puncture, not transfer position change, significantly increased the average number of live piglets and cloning efficiency.

Normal transfer referred to that cloned embryos were transferred to the oviduct ampulla, which is about 10 cm away from umbrella; follicle puncture was that all the large developed follicles were punctured when surrogate gilts under preovulation; and, transfer position shallowed or deepened was that transfer position was about 8 cm or 12 cm away from umbrella.

The rates of pregnancy and delivery and the average numbers of piglets born and alive were based on the number of surrogates.

Cloning efficiency was calculated by total number of live cloned piglets/total number of transferred cloned embryos.

^a,b^Values in the same column with different superscripts differ significantly (P < 0.05).

## Discussion

In the previous study, we have cloned several excellent pigs [3]. In order to accelerate the large-scale multiplication of superior genotypes, here, we systematically investigated the major influencing factors including donor cell type, number of cloned embryos transferred per surrogate, surrogate estrus stage and ovulation status related to the overall cloning efficiency to improve the generation of cloned pigs. In this study, a total of 13620 cloned embryos were transferred into 79 surrogates, 119 live cloned piglets with superior genotypes were eventually generated, and cloning efficiency was 0.93%. And, we also found that surrogate ovulation status was the fundamentally decisive factor influencing the overall cloning efficiency.

SCNT has been widely applied to clone genetically superior pigs [4]. Nevertheless, to date, the overall cloning efficiency is still low, and this limits the practical application of pig SCNT technique in swine industry and bioscience research [8]. It is known that cloning is a complex multistep progress, and many potential factors are believed to influence SCNT efficiency [10]. Donor cell type is considered to be one key factor [11]. Currently, it is still unclear that which donor cell type is most suitable for pig cloning. Generally, fetal fibroblast cells are usually used to generate cloned pigs [14]. However, in this study, only PAFs were applied, as we aimed to clone excellent pigs. Our results showed that L-PAFs and LW-PAFs had a similar overall cloning efficiency, and this cloning efficiency is basically consistent with the reported cloning efficiency derived from fetal fibroblasts [21]. And, different cell lines of PAFs also displayed no significant differences of in vitro development of cloned embryos (Table D in S1 File). Thus, donor cell types examined could be not considered to be a major factor influencing the cloning efficiency. Of course, other donor cell types derived from excellent pigs could probably influence cloning efficiency, as the link between donor cell type and the developmental competence of cloned embryos is complex [22], however, these donor cell types are not available as excellent pigs are still in use. Maybe, when they are eliminated, we can obtain various cell types and apply these donor cells to SCNT, thereby further clarifying the relationship between donor cell type and the developmental potential of cloned embryos.

The number of cloned embryos transferred per surrogate is also an issue influencing the cloning efficiency. Previous studies have shown that establishment and maintenance of pig pregnancy need at least 4 high quality blastocysts, and surrogate pregnancy and delivery rates are associated with the number of transferred cloned embryos [11, 13, 21, 23], thus, in view of the poor developmental ability of porcine cloned embryos, a large amount of cloned embryos are usually transferred into surrogates. In this study, no significant differences of the overall cloning efficiency were observed among the 101–150, 151–200 and 201–250 groups. It is possible that the number of cloned embryos transferred has met the requirement for full term development. Though the ability of cloned embryos developing to blastocysts in vivo is not known, in vitro development of cloned embryos could lead to about 20% blastocyst rate, suggesting that there would be about 20 blastocysts even for the minimal number of cloned embryos transferred in this study. Thus, approximately 100 cloned embryos transferred per surrogate is enough to give birth to cloned piglets. And, uterine space is limited, not allowing supernumerary fetuses to develop to term, so transfer of excessive cloned embryos per surrogate is not necessary to increase the overall cloning efficiency. Moreover, less than 100 cloned embryos could generate cloned piglets, and only 15 cloned embryos at the 2–4 cell stage has produced live cloned pigs [24, 25]. Therefore, 101–150, 151–200 or 201–250 cloned embryos are enough to generate cloned pigs, and how many cloned embryos transferred per surrogate is appropriately suitable needs further investigation.

Surrogate status has been considered as an important factor affecting the outcome of pig SCNT [9]. It is known that the synchronization between cloned embryos and surrogates determines the subsequent development, and the primary cause of failure in the development of cloned embryos transferred is that cloned embryos do not match the oviduct environment [12]. Here, we transferred cloned embryos at 14 h postactivation into surrogates with different estrus stage to coordinate the synchronization, and the results showed that surrogates with estrus on Day 2 was beneficial for the implantation and full term development of cloned embryos. The probable cause is that cloned embryos developed at a slower rate than their in vivo counterparts and were just in synchronization with surrogates with estrus on Day 2 when cultured 14 h postactivation [26]. In addition, most surrogates in the Day 2 group were under periovulation, and nearly all surrogates in the periovulation group promoted the full term development of cloned embryos and gave birth to cloned pigs. Also, surrogate ovulation status has been reported to influence the generation of cloned pigs [12]. Thus, surrogate ovulation status is a decisive factor influencing the generation of cloned pigs. Moreover, the extent of synchronization for preovulation and postovulation surrogates was also considered, and transfer position change did not improve the overall cloning efficiency, seeming that cloned embryos at some stage should be transferred to a certain position of surrogate oviduct. Interestingly, follicle puncture improved cloning efficiency in the preovulation group. This result suggests that follicle puncture probably adjusted the physiological environment in the oviduct and made it suitable for the development of cloned embryos cultured 14 h postactivation. Overall, when surrogate estrus was 28 h prior to cloned embryo postactivation, and cloned embryos cultured 14 h postactivation were transferred to the surrogate ampulla, the oviduct environment was adequate and supported the subsequent development of cloned embryos. Nevertheless, the synchronization between cloned embryos and surrogates, including estrus stage, transfer time, transfer position, embryo stage, et al., is complicated, and the optimal synchronization manner remains to be studied.

It is known that animal cloning is a complex progress, and many other potential factors, embryo quality for example, should also be investigated [24]. In this study, we found that a large proportion of cloned piglets died when birth or during growth. This may be due to incomplete reprogramming of SCNT embryos, leading to the abnormality of cloned pigs [1]. Thus, methods to improve embryo quality would be applied in the further pig cloning [27]. Importantly, it is observed that surrogate ovulation status is associated with the overall cloning efficiency, so, the strategy to coordinate the synchronization between cloned embryos and surrogates in the further pig cloning, such as ovulation detection by ultrasonography, would be particularly important. Whatever, we believe that investigating and regulating factors related to SCNT would improve cloning efficiency and enlarge the practical application of SCNT, further promoting the development of swine industry and bioscience research.

In conclusion, we have successfully cloned a number of excellent pigs, and our results displayed that selecting surrogate gilts with estrus on Day 2 and under periovulation to transfer cloned embryos at 14 h postactivation was beneficial for the subsequent development of cloned embryos, and follicle puncture, not transfer position change, improved cloning efficiency. We recommended that surrogate ovulation status was the fundamental factor determining the overall cloning efficiency, and all the strategies to enhance the full term development of embryos should always take the synchronization between donor embryos and surrogates into consideration.

## Supporting Information

S1 Fig The detail number of surrogate gilts applied in the investigation of the effects of factors on pig cloning. A total of 79 surrogate gilts were investigated to assess the effect of individual factor on the overall cloning efficiency, among which, 57 surrogates were used to assess the effect of normal transfer (no operation to transfer position and follicle) including donor cell type, number of cloned embryos transferred per surrogate, surrogate estrus stage and ovulation status, and 58 surrogates containing 15 preovulation normally transferred, 21 postovulation normally transferred, 13 transfer position changed and 9 follicle punctured were investigated to analyze the effect of transfer manner. Notably, the extra 36 surrogates were the 15 preovulation and 21 postovulation surrogates in the normal transfer group, and also the control in the transfer manner changed group.
(TIF)

S2 Fig Donor cells, corresponding cloned blastocysts and blastocyst cell numbers. A, A' and A'': L-PAFs (× 200) and their corresponding cloned blastocysts (× 40) and blastocyst cell numbers (× 200), and B, B' and B'': LW-PAFs (× 200) and their corresponding cloned blastocysts (× 40) and blastocyst cell numbers (× 200). L-PAFs and LW-PAFs groups displayed no significant differences of in vitro development of cloned embryos.
(TIF)

S3 Fig The cloned piglets. A, cloned piglets derived from Landrace breeding boars, and B, cloned piglets derived from Large white breeding boars. Cloned Landrace and Large white piglets were successfully generated.
(TIF)

S4 Fig Transfer position and follicle puncture. Follicle puncture not transfer position change significantly increased the average number of live piglets and cloning efficiency. Transfer position referred to that cloned embryos were transferred to the oviduct ampulla, or the position of about 8 cm or 12 cm in the oviduct away from umbrella, and follicle puncture was that all the large developed follicles were punctured with the needle of 1ml syringe when surrogate gilts under preovulation.
(TIF)

S1 File The detail content of Table A-D. Table A, effect of donor cell types on in vitro development of cloned embryos. Table B. Multivariate tests of surrogate estrus stage and ovulation status on the overall cloning efficiency. Table C, in vivo development of cloned embryos transferred into surrogates in the Day 2 group and under periovulation. Table D, effect of different cell lines of PAFs on in vitro development of cloned embryos. **Table A Effect of donor cell types on in vitro development of cloned embryos.** The detail of numbers of embryos, fused embryos, cleaved embryos and blastocysts and blastocyst cell numbers in the L-PAFs and LW-PAFs groups. **Table B Multivariate tests of surrogate estrus stage and ovulation status on the overall cloning efficiency. The detail result of multivariate tests of estrus stage and ovulation status. Table C The overall cloning efficiency of surrogates in the Day 2 group and under periovulation.** The detail numbers of surrogates, embryos transferred, pregnancy, delivery, piglets born, piglets alive and cloning efficiency in the Day 2 group and under periovulation. **Table D In vitro development of cloned embryos derived from different cell lines of PAFs.** The detail of numbers of embryos, fused embryos, cleaved embryos and blastocysts and blastocyst cell numbers in the groups of two different cell lines.
(DOC)
