# Supplementary figures and images for: Ovulation Statuses of Surrogate Gilts Are Associated with the Efficiency of Excellent Pig Cloning
Source: PLoS One. 2015 Nov 13;10(11):e0142549. doi: 10.1371/journal.pone.0142549 (PMC4643933; doi:10.1371/journal.pone.0142549)

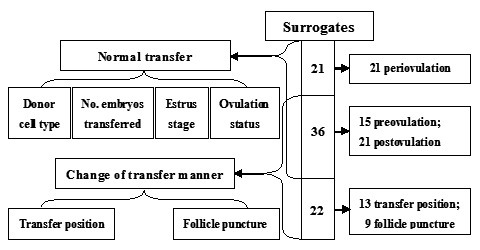

Supplement: S1 Fig — A total of 79 surrogate gilts were investigated to assess the effect of individual factor on the overall cloning efficiency, among which, 57 surrogates were used to assess the effect of normal transfer (no operation to transfer position and follicle) including donor cell type, number of cloned embryos transferred per surrogate, surrogate estrus stage and ovulation status, and 58 surrogates containing 15 preovulation normally transferred, 21 postovulation normally transferred, 13 transfer position changed and 9 follicle punctured were investigated to analyze the effect of transfer manner. Notably, the extra 36 surrogates were the 15 preovulation and 21 postovulation surrogates in the normal transfer group, and also the control in the transfer manner changed group. (TIF) [file pone.0142549.s001.tif]

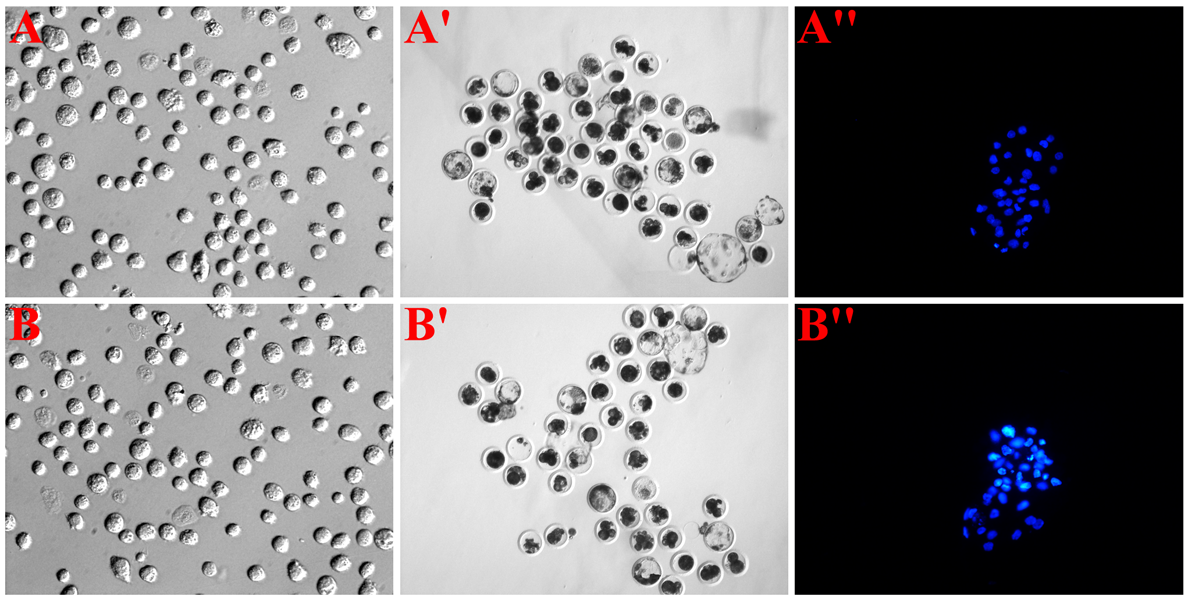

Supplement: S2 Fig — A, A' and A'': L-PAFs (× 200) and their corresponding cloned blastocysts (× 40) and blastocyst cell numbers (× 200), and B, B' and B'': LW-PAFs (× 200) and their corresponding cloned blastocysts (× 40) and blastocyst cell numbers (× 200). L-PAFs and LW-PAFs groups displayed no significant differences of in vitro development of cloned embryos. (TIF) [file pone.0142549.s002.tif]

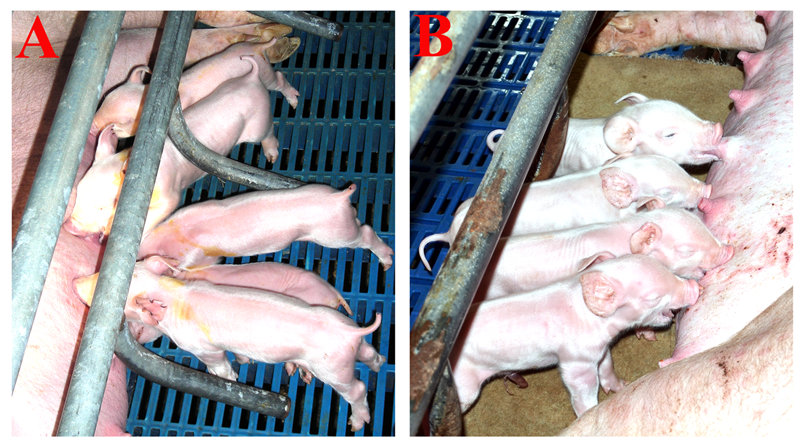

Supplement: S3 Fig — A, cloned piglets derived from Landrace breeding boars, and B, cloned piglets derived from Large white breeding boars. Cloned Landrace and Large white piglets were successfully generated. (TIF) [file pone.0142549.s003.tif]

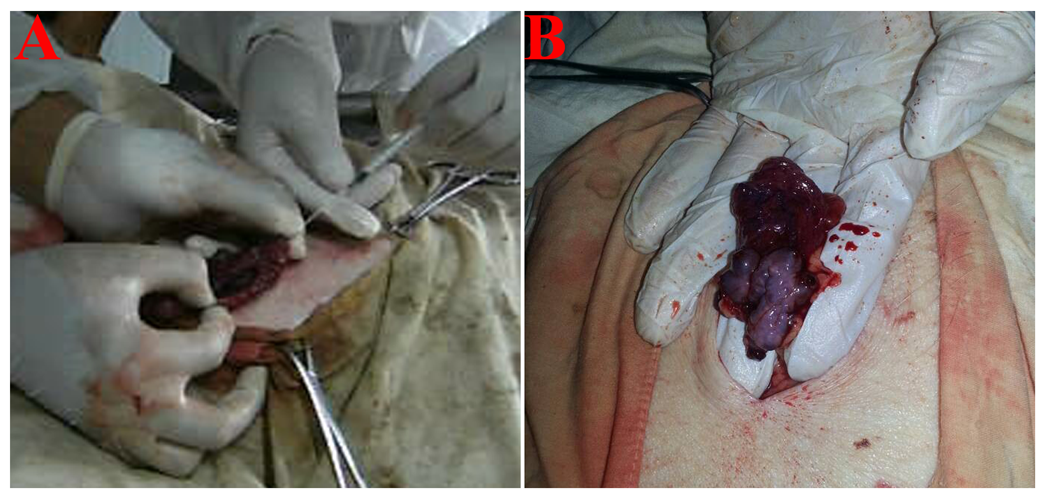

Supplement: S4 Fig — Follicle puncture not transfer position change significantly increased the average number of live piglets and cloning efficiency. Transfer position referred to that cloned embryos were transferred to the oviduct ampulla, or the position of about 8 cm or 12 cm in the oviduct away from umbrella, and follicle puncture was that all the large developed follicles were punctured with the needle of 1ml syringe when surrogate gilts under preovulation. (TIF) [file pone.0142549.s004.tif]
