# Supplementary material for: Ovulation Statuses of Surrogate Gilts Are Associated with the Efficiency of Excellent Pig Cloning
Source: PLoS One. 2015 Nov 13;10(11):e0142549. doi: 10.1371/journal.pone.0142549 (PMC4643933; doi:10.1371/journal.pone.0142549)
Supplement: S1 File — Table A, effect of donor cell types on in vitro development of cloned embryos. Table B. Multivariate tests of surrogate estrus stage and ovulation status on the overall cloning efficiency. Table C, in vivo development of cloned embryos transferred into surrogates in the Day 2 group and under periovulation. Table D, effect of different cell lines of PAFs on in vitro development of cloned embryos. Table A Effect of donor cell types on in vitro development of cloned embryos. The detail of numbers of embryos, fused embryos, cleaved embryos and blastocysts and blastocyst cell numbers in the L-PAFs and LW-PAFs groups. Table B Multivariate tests of surrogate estrus stage and ovulation status on the overall cloning efficiency. The detail result of multivariate tests of estrus stage and ovulation status. Table C The overall cloning efficiency of surrogates in the Day 2 group and under periovulation. The detail numbers of surrogates, embryos transferred, pregnancy, delivery, piglets born, piglets alive and cloning efficiency in the Day 2 group and under periovulation. Table D In vitro development of cloned embryos derived from different cell lines of PAFs. The detail of numbers of embryos, fused embryos, cleaved embryos and blastocysts and blastocyst cell numbers in the groups of two different cell lines. (DOC) [file pone.0142549.s005.doc]

**Table A. Effect of donor cell types on in vitro development of cloned embryos.**

| **Cell types** | **No. embryos (Rep.)** | **No. embryos fused (% ± SEM)** | **No. embryos cleaved (% ± SEM)** | **No. blastocysts (% ± SEM)** | **Total cell numbers of blastocysts (mean ± SEM)** |
| --- | --- | --- | --- | --- | --- |
| **L-PAFs** | 293 (5) | 254 (86.46 ± 0.92) | 222 (87.40 ± 0.56) | 48 (18.48 ± 0.38) | 38 ± 2 (n = 46) |
| **LW-PAFs** | 282 (5) | 239 (84.53 ± 1.13) | 209 (87.63 ± 0.99) | 47 (19.73 ± 0.71) | 39 ± 3 (n = 46) |

No significant differences of in vitro development were observed between the L-PAFs and LW-PAFs groups.

Cleavage and blastocyst rates were adjusted for fusion rates.

Blastocyst cell numbers, less than 16, were not included.

**Table B. Multivariate tests of surrogate** **estrus stage and ovulation status on the overall cloning efficiency.**

| **Effect** | **Test** | **Value** | **F** | **Hypothesis df** | **Error df** | **Sig.** |
| --- | --- | --- | --- | --- | --- | --- |
| **Estrus** | Pillai's Trace | 0.27 | 0.72 | 20.00 | 196.00 | 0.81 |
|  | Wilks' Lambda | 0.75 | 0.70 | 20.00 | 153.52 | 0.83 |
|  | Hotelling's Trace | 0.30 | 0.68 | 20.00 | 178.00 | 0.85 |
|  | Roy's Largest Root | 0.14 | 1.37 | 5.00 | 49.00 | 0.25 |
| **Ovulation** | Pillai's Trace | 0.47 | 2.92 | 10.00 | 94.00 | 0.00 |
|  | Wilks' Lambda | 0.56 | 3.07 | 10.00 | 92.00 | 0.00 |
|  | Hotelling's Trace | 0.71 | 3.22 | 10.00 | 90.00 | 0.00 |
|  | Roy's Largest Root | 0.61 | 5.73 | 5.00 | 47.00 | 0.00 |

Ovulation status was the real factor underlying estrus stage to determine the overall cloning efficiency.

The results of Multivariate tests differ significantly (P < 0.05).

**Table C. I**n vivo development of cloned embryos transferred into surrogates in the Day 2 group and under periovulation.

| **No. surrogates** | **No. embryos transferred (mean ± SEM)** | **No. pregnancy (%)** | **No. delivery (%)** | **No. piglets (mean ± SEM)** | **No. piglets alive (mean ± SEM)** | **Cloning efficiency (%)** |
| --- | --- | --- | --- | --- | --- | --- |
| 14 | 2260 (161.43 ± 9.37) | 14 (100.00 ± 0.00) | 14 (100.00 ± 0.00) | 70 (5.00 ± 0.69) | 49 (3.50 ± 0.50) | 2.20 ± 0.32 |

Surrogates under periovulation in the Day 2 group displayed a very high overall cloning efficiency.

The rates of pregnancy and delivery and the average numbers of piglets born and alive were based on the number of surrogates.

Cloning efficiency was calculated by total number of live cloned piglets/total number of transferred cloned embryos.

**Table D. Effect of different cell lines of PAFs on in vitro development of cloned embryos.**

| **Cell line** | **No. embryos (Rep.)** | **No. embryos fused (% ± SEM)** | **No. embryos cleaved (% ± SEM)** | **No. blastocysts (% ± SEM)** | **Total cell numbers of blastocysts (mean ± SEM)** |
| --- | --- | --- | --- | --- | --- |
| 1 | 293 (5) | 251 (85.69 ± 0.93) | 219 (87.24 ± 1.17) | 52 (20.99 ± 1.18) | 37 ± 2 (n = 52) |
| 2 | 318 (5) | 266 (83.88 ± 1.94) | 233 (87.76 ± 0.75) | 53 (20.08 ± 0.89) | 38 ± 3 (n = 52) |

No significant differences of in vitro development were observed between these two different cell lines.

Cleavage and blastocyst rates were adjusted for fusion rates.

Blastocyst cell numbers, less than 16, were not included.
